# Detection of spatial avoidance between sousliks and moles by combining field observations, remote sensing and deep learning techniques

**DOI:** 10.1038/s41598-022-12405-z

**Published:** 2022-05-18

**Authors:** Rafał Łopucki, Daniel Klich, Piotr Kociuba

**Affiliations:** 1 Department of Biomedicine and Environmental Research, The John Paul II Catholic University of Lublin, Konstantynów 1J, 20-708 Lublin, Poland; 2 Department of Animal Genetics and Conservation, Warsaw University of Life Sciences-SGGW, Ciszewskiego 8, 02-786 Warsaw, Poland; 3 Institute of Mathematics, Informatics and Landscape Architecture, The John Paul II Catholic University of Lublin, Konstantynów 1H, 20-708 Lublin, Poland

**Keywords:** Conservation biology, Restoration ecology

## Abstract

Nowadays, remote sensing is being increasingly applied in ecology and conservation, and even underground animals can successfully be studied if they leave clear signs of their presence in the environment. In this work, by combining a field study, analysis of high-resolution aerial images, and machine learning techniques, we investigated the interspecies relationships of two small burrowing mammals: the spotted souslik *Spermophilus suslicus* and the European mole *Talpa europaea*. The study was conducted for 3 years (2018–2020) at a 105-ha grass airfield where both species coexist (Poland). Both field studies and the analysis of aerial imagery showed that, in the period of low population numbers, the souslik avoided coexistence with the European mole, and the presence of the mole was found to reduce the area of the habitat suitable for the souslik. The presence of other burrowing species may be an important element in the habitat selectivity of the souslik, but this has not yet been included in the conservation guidelines for this species. We discuss the contribution of our results to the knowledge of the ecology of burrowing mammals and their interspecies relationships. We also assess the possibility of using remote sensing and deep learning methods in ecology and conservation of small burrowing mammals.

## Introduction

Nowadays, remote sensing is being increasingly applied in ecology and conservation^1–4^. These techniques facilitate not only mapping habitats^5^, but also investigating populations of some animal species and their temporal changes^6–8^. However, the applicability of techniques based on satellite or aerial imagery to study animals is strongly influenced by the body size of the species studied and their visible or cryptic lifestyle. If the animals are large enough and the habitat does not mask their visibility from the air, very detailed remote population surveys can be performed (e.g.^6^ for elephant seal or^8^ for African elephants).

Obviously, the biggest challenge for remote sensing research is posed by small secretive animals, especially underground species. Nevertheless, effective research of such animals can be carried out. These studies do not aim at direct observation of animals but focus on specific and clear signs of their presence in the environment. Studies conducted using aerial or satellite images for the detection of termite mounds^9^, vole and lemming activity^10^, wombat burrows^11^, or American beaver range expansion^7^ are some examples of such research. On a geographical scale (area of 950,000 km^2^), a similar study was also done for the Bobak marmot^12^. Using mounds as an indicator of the occurrence of the species, the current population size for Kazakhstan was estimated and it was indicated that the range of this species had remained almost unchanged since the 1950s, despite the few drastic episodes of land-use change. This study challenged the opinion that the population has decreased due to agricultural expansion. Based on these results, Koshkina et al.^12^ suggest that, since the coverage and availability of free high-resolution satellite images increase, a similar approach should be transferable to many burrowing mammals with a sufficient size of burrows or mounds.

In our paper, we follow the suggestions made by Koshkina et al.^12^ and we test the possibility of using high-resolution images to study spatial relationships in two European small burrowing mammals: semi-fossorial spotted sousliks *Spermophilus suslicus* (Güldenstaedt, 1770) and fossorial moles *Talpa europaea* (Linnaeus, 1758). Both species weigh less than 500 g and spend most of their life underground. The terrestrial signs of their presence are small mounds of soil resulting from digging or maintaining burrows. These species, however, are radically different in their conservation status. The European mole is a common, widespread, eurytopic species for which no specific conservation measures are recommended (http://www.iucnredlist.org/species/41481/22320754). On the other hand, the spotted souslik is an extremely endangered species in the European Union and is known from only one relict enclave in south-eastern Poland^13–15^. The souslik population has been declining since the 1950s, and despite the protective measures taken, this negative trend has not been reversed^15^. This species and its habitats as either nature reserves or Natura 2000 sites are protected by law at the European and national levels^16,17^.

So far, the spatial relationships between the mole and the souslik have not been studied in detail, and other parameters, mainly soils and vegetation, have been taken into account when assessing the optimal souslik habitat^14,18,19^. Thus, it is not known whether the occurrence of other burrowing species affect the habitat preferences of the souslik and may be a significant factor limiting or supporting the population growth of this endangered species. Literature data indicate that in the period of small population size, especially after reintroductions, the souslik may have specific habitat requirements differing from those described for stabilized and abundant populations^20^. It has also been reported that unexpected interspecies interactions may sometimes be important for the habitat preferences of the souslik during the colonization of a new area, e.g. in Bulgaria, *Lasius flavus* anthills were chosen by the souslik as preferred places to locate burrows^21^. It can therefore be expected that the presence of another mammalian burrowing species, such as the mole, may also affect the souslik, especially in critical periods of low population abundance. It has already been shown in the literature that the presence and density of the mole can significantly affect other burrowing species. For example, strong interrelationships have been demonstrated between the mole and the European water vole *Arvicola amphibius*^22^.

Our work combined a field study, analysis of high-resolution aerial imagery, and machine learning techniques. We checked (1) whether spatial pattern of the European mole and the spotted souslik co-occurrence is random or non-random, and if the latter, whether there is spatial avoidance or preference between these species, and (2) whether aerial remote sensing could be effectively used to study and predict the distribution of these small burrowing mammals. We assumed our results to prove that aerial remote sensing can be used in non-invasive detection of small burrowing mammals. We also expected that the results of this study could contribute to better conserving sousliks through better understanding its habitat preferences influenced by another burrowing mammal.

## Methods

### Study area

**Figure 1:**
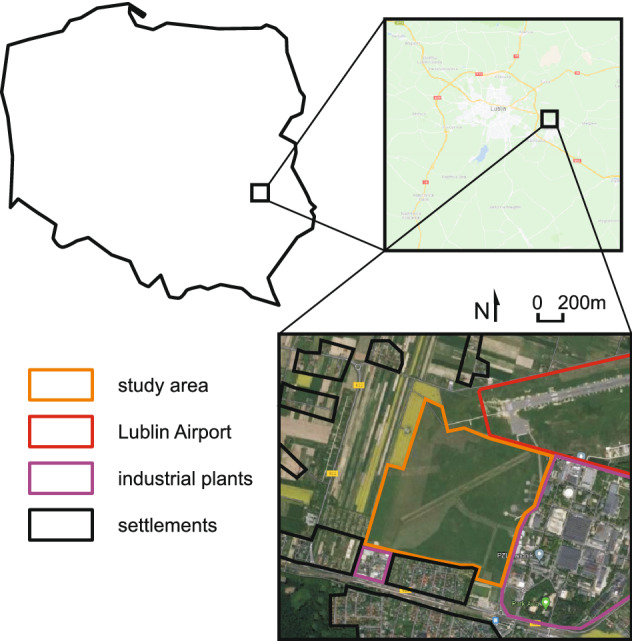
Location of the study area in Poland (Europe), the shape of the grass airfield, and its surroundings. The figure was generated in Google Earth Pro v7.3 (https://earth.google.com) and CorelDRAW Standard 2020 (https://www.corel.com).

The study was carried out in a spotted souslik colony located at a grass airfield in Świdnik near Lublin (Poland, airfield coordinates: 51.232081°N, 22.690224°E). The study area covered approximately 105 ha with short vegetation (grasses and herbs) surrounded mainly by industrial areas and settlements (Fig. 1). The airfield area is regularly mowed both for the safety of aeroplanes and to maintain favourable conditions for the spotted souslik.

The entire airfield area is protected under the Natura 2000 network, i.e. Special Protection Area PLH060021. The Natura 2000 area in Świdnik is one of the largest protected areas established for the conservation of the spotted souslik in Poland. At the beginning of the twenty-first century, approx. 90% of the Polish souslik population (even several thousand individuals) lived in this colony^15^. Currently, the number of the spotted souslik in Poland is low, and the population in Świdnik in the years preceding the study and in the years of the study was about 200 individuals (detailed number in late summer: 2016–231; 2017–226; 2018–229; 2019–72; 2020–71). Such a population size meant that sousliks were not limited by overcrowding and could theoretically occupy any fragments of this extensive habitat.

### Field study

In this study we did not use of experimental animals and did not capture the wild animals, so we did not need the approval of the ethics committee. The fields methods used in this study were carried out in accordance with conservation guidelines for the spotted souslik^14^ and were approved by the Regional Director of Environmental Protection in Lublin. We conducted a 3-year field study to collect material for validation of the results of remote detection of mole mounds using aerial images and machine learning. During the field study, each year, we located the nest burrows of all spotted sousliks living in the study area and the location of the mole mounds in relation to the souslik burrows according to the methodology described below.

#### Field study of the spotted souslik

The study of the distribution and size of the spotted souslik population was carried out in spring (April–May) for three consecutive years, 2018–2020. A commonly used indicator for determining the number of sousliks is the monitoring of their burrows, and the principles of such monitoring are well described in the conservation guidelines^14^. Briefly, monitoring is carried out in two steps. First, trained observers inspect the entire area on foot and mark any souslik burrows found using a GPS receiver and/or physical markers (e.g. small flags). After all the burrows have been marked, the next step is to find out which of the burrows are inhabited by sousliks. For this purpose, early in the morning the entrances to the burrows found are blocked (using hay or straw) and, after a few hours, the observers check which burrows have been reopened. Since only one souslik inhabits one burrow in spring, it is assumed that the number of reopened burrows is a reliable indicator of the population size^14,23^. In comparative studies, this method was found to be the simplest and most reliable to assess the number and distribution of sousliks^20^. It is also important that this method does not require trapping and marking animals, hence it is minimally invasive. After determining the final number of inhabited souslik burrows, a layer was prepared in the QGIS program showing the distribution and number of souslik colonies in the study area in a given year.

#### Field study of the European mole occurrence

The occurrence of the mole was considered in the context of its co-occurrence with the souslik. For this reason, signs of the mole's presence (molehills) were searched for in three different zones into which the study area was divided.

1. The first zone (BURROW) was marked out near the sousliks’ burrows. For this purpose, a circle with an area of 10 m^2^ (3.56 m in diameter) was established around the burrow inhabited by the souslik (in spring, the burrow includes a nest chamber with a vertical corridor bent at one point at an angle of about 45°, so if we determine a circle with an area of 10 m^2^ around the entrance to the burrow, the underground nest chamber will probably be located within it). On the plot determined in this way, the presence or absence of mole mounds was checked and recorded in a binary form: 1—mole mounds present; 0—no mole mounds. The number of measurements made in this zone was 91, 200, and 84 in 2018, 2019, and 2020, respectively.
2. The second zone (COLONY) was the area of the souslik colony, but without taking into account the areas near the burrows. To determine this zone, data on the location of souslik burrows in a given year were used and the area of the souslik colony with kernel density estimation of 95% of all locations was determined in the QGIS software. Then, the areas near the burrows up to a distance of 4 m were cut out (excluded) to limit the autocorrelation with the results from the first zone. In such a designated area, 100 random points were determined each year. During the drawing of the points, it was established that the distance between them must not be less than 8 m to avoid spatial overlapping of adjacent surfaces. The coordinates of each of the drawn points were uploaded to a GPS receiver and the randomly selected points were found in the field. A circle with an area of 10 m^2^ was marked around each point and it was checked whether there were any mole presence signs and the results were recorded as “presence” or “absence”.
3. The third zone (CONTROL) was the remaining area of the airfield, outside the areas of the souslik colony. In this zone, points were also randomly determined in the QGIS program, ensuring that the distances between them were not less than 8 m to avoid spatial overlapping of adjacent plots. The number of designated points was as follows: 150 points in 2018, 120 points in 2019, and 200 points in 2020. Then, the points were uploaded to a GPS receiver (Montana 680t) and found in the field with accuracy up to 1 m. A circle of 10 m^2^ was drawn around each of the points and signs of the mole presence were noted using the binary presence/absence codes.

### Remote detection of mounds using aerial images and machine learning

#### Mole mounds in aerial images

**Figure 2:**
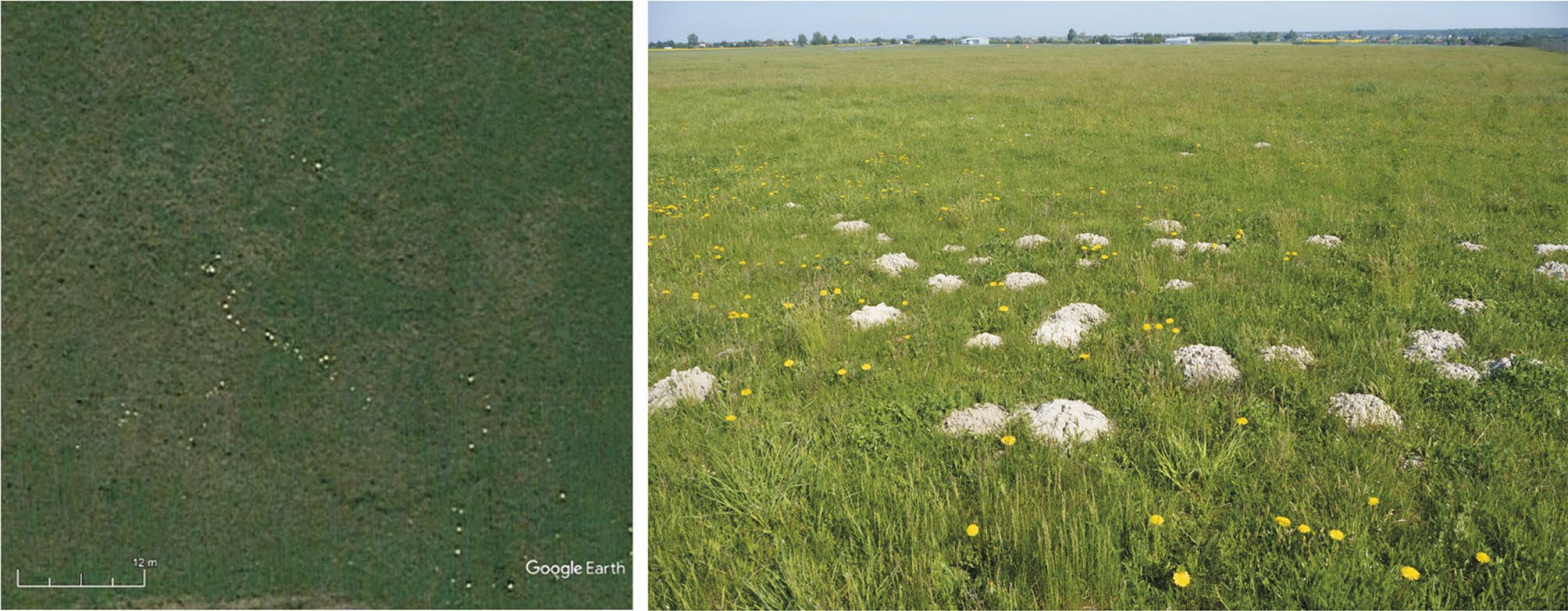
Examples of mole mounds in the study area (May 5, 2017) are visible even in low-resolution photos made available in Google Earth as bright points of loess soil against the darker vegetation of the airport grassland. The figure was generated in Google Earth Pro v7.3 (https://earth.google.com) and CorelDRAW Standard 2020 (https://www.corel.com).

**Figure 3:**
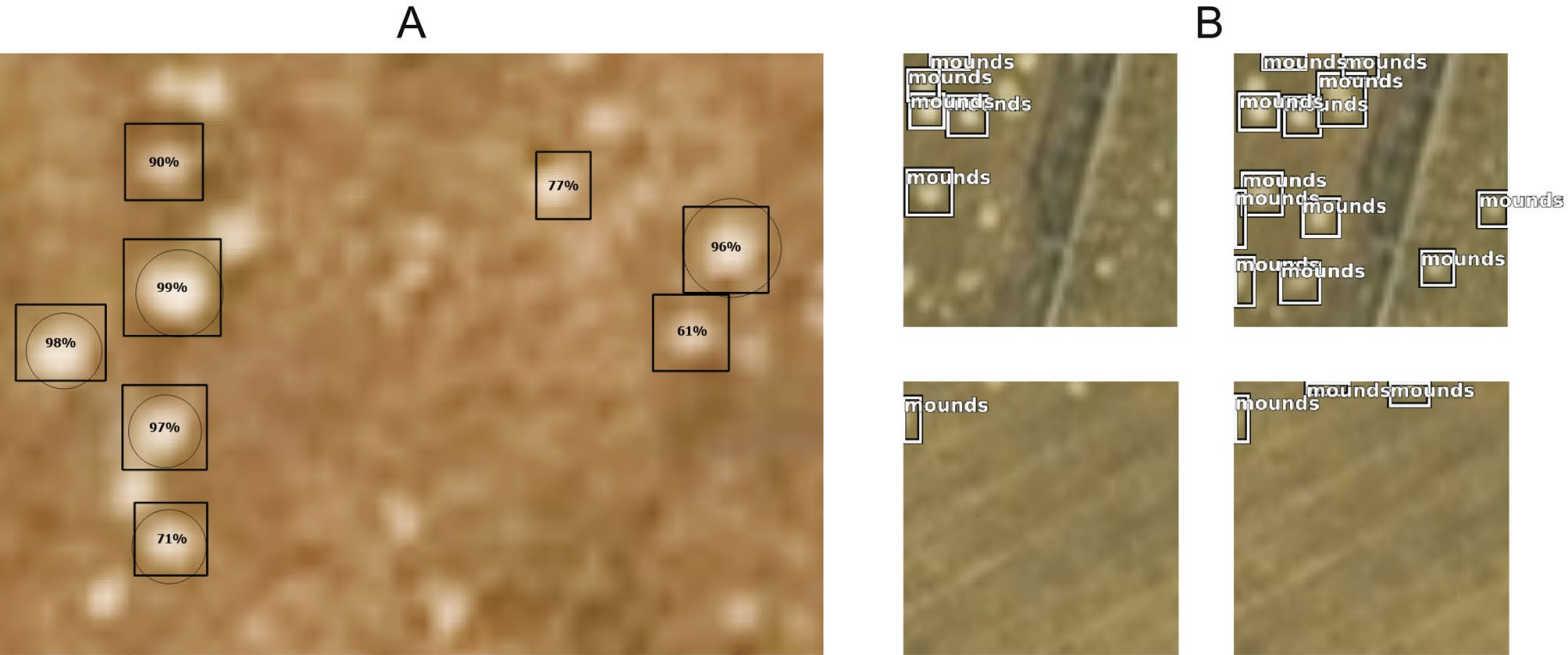
Examples of labelled mounds, precision score (**A**), and final results of mound recognition in four randomly selected sites (**B**). The figure was generated in ArcGIS Pro v2.8 (https://www.esri.com) and CorelDRAW Standard 2020 (https://www.corel.com).

Using aerial images, we focused on the detection of signs of the presence of moles, specifically the detection of mole mounds. Mole mounds are about 15–50 cm in diameter. In the aerial images from our study area, they are visible as light spots of loess soil on a darker matrix of short vegetation (Figs. 2, 3).

There are no other burrowing animal species in the study area that could form similar mounds. The mounds left by the souslik have a different elongated shape and are much larger (they can be even more than 1 m in length). The ground layer made by the souslik is thinner, which is why it is quickly overgrown by vegetation^14,24^. In addition, sousliks dig their burrows only at a certain time of the year (late spring and summer); hence, in early spring, there are no mounds of soil made by the souslik that could be mistaken for mole mounds in aerial images. Another rodent species present in the study area, i.e. the common vole *Microtus arvalis*, does not form distinct mounds, and the external appearance of the burrows of this species is different from mole burrows. In the Polish fauna, the European water vole *Arvicola amphibius* produces mounds of soil similar to the mole. However, the presence of this species can be excluded, as it prefers mainly wet habitats. We also made sure that no anthropogenic activities (such as digging in the ground) that could have led to creation of objects similar to mole mounds were carried out in the study area.

#### Aerial imagery and processing

For detection of mole mounds, we used a free orthoimagery dataset from GUGiK (http://www.gugik.gov.pl) downloaded from the geoportal site (http://www.geoportal.gov.pl). This dataset offers aerial imagery with a resolution of 25 cm. We downloaded sheets M-34-34-A-d-1-2, M-34-34-A-d-2-1, M-34-34-A-d-2-3, and M-34-34-A-d-1-4 (acquisition date: 15.03.2020). We resampled orthoimagery and changed the cell size to 2 cm with the bilinear resampling method in ArcGIS Pro using the “Resample” tool. Image interpolation algorithms are widely used in a large set of devices and applications, such as medical image processing tools, where the input is often a noisy, low resolution image (e.g.^25^). This concept allowed the use of an interpolation algorithm to obtain a better resolution orthoimagery for training the deep learning model.

**Figure 4:**
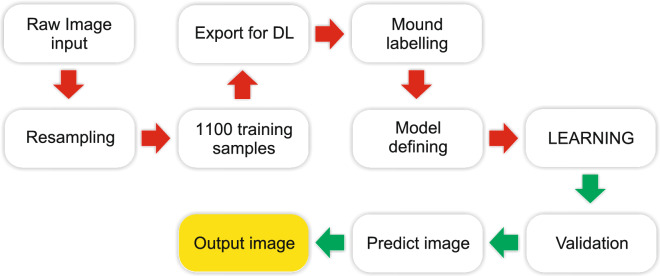
Diagram of the mound detection network. The figure was generated in CorelDRAW Standard 2020 (https://www.corel.com).

**Figure 5:**
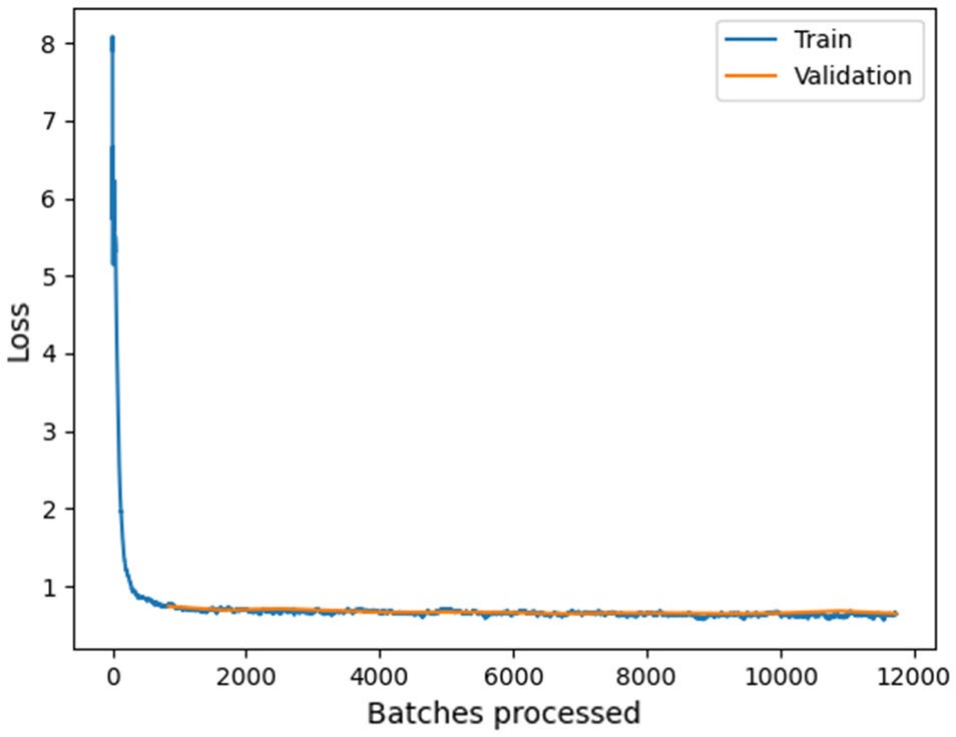
Training losses and validating losses over training batches. The figure was generated in ArcGIS Pro v2.8 (https://www.esri.com).

In ArcGIS Pro, 1100 training samples were created using the “Label Object for Deep Learning” tool. This software includes the “Export Training Data for Deep Learning” tool that can be used to create labelled images needed to train a deep learning model. Based on this method, we created 1987 images with a resolution of 256 × 256 pixels, where known mounds in the images were labelled (Figs. 3, 4). The test set consisted of approximately 30% of all images. We used TensorFlow deep learning libraries with Faster-RCNN^26^ and backbone resnet101^27,28^ for approximately 12,000 training steps in total with a learning rate of 0.001. Figure 5 presents the loss curves of training and validating during the training. The model training was stopped when the model was no longer improving. The following metrics were used to evaluate the classification model: precision (TP/(TP + FP)), recall (TP/(TP + FN) and F1-score^29^ with “Compute Accuracy For Object Detection” tool in ArcGIS Pro. True Positives [TP] is the number of detections with intersection over union (IoU) > 0.5. False Positives [FP] is the number of detections with IoU ≤ 0.5 or detected more than once. False Negatives [FN] is the Number of objects that not detected or detected with IoU ≤ 0.5. F1-score is HM (Harmonic Mean) of precision and recall (2TP/(2TP + FP + FN)).

### Comparison of field study results and remote mound detection

The machine learning procedures described above showed that the average precision score in recognition of mole mounds by the analyzed aerial imagery was 0.6845. We checked whether this precision in detecting mole burrows generated results consistent with the results of fieldwork. To this end, we used ArcGIS Pro software and put all the 10m^2^ plots on the aerial imagery of the study area, in which we assessed the presence or absence of mole mounds in 2019 (200, 100, and 120 plots from zones 1, 2, and 3, respectively). Then, we checked on how many of these plots mole mounds were detected by automatic image processing. We presented the obtained results in the same way as for the field studies, i.e. as a proportion of plots with mole mounds in a given zone.

We also compared the area of the airfield grassland available to the souslik without taking into account the presence of moles (theoretically 105 ha) and after taking this factor into account. We used data on the distribution of the mole based on aerial imagery and determined a centroid for each mole mound. Then, using these points, the area occupied by the mole was determined with the kernel density estimation (95%) method. To determine the boundaries of this area, we assumed two different radii: 5 and 10 m. This made it possible to calculate the area of the habitat unoccupied by other burrowing species that could potentially affect the spotted souslik.

### Statistical analysis

We collected both geographic data (location information) and quantitative data, which we analyzed statistically. Our field study data matrix included information on the number of study plots where the mole mounds were found in each of the three study zones and each study year. The frequency of the mole mounds in each study zone was compared separately for each study year, and the method of generalized linear binary models, where the frequency of the mole mounds was always a dependent variable, was used. An independent variable in each model was the study zone, with the three categories mentioned above (BURROW, COLONY, CONTROL). The models were evaluated with the area under the ROC curve (AUC). The pairwise comparison between the categories was performed with the LSD test. All statistical analyses were performed using SPSS software (version 24.0, IBM Corporation, Armonk, NY, USA).

## Results

### Field study

**Figure 6:**
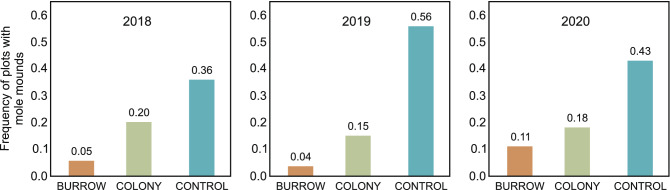
Frequency of plots with mole mounds in the three studied zones presented separately for each year of the study. In each graph: BURROW—plots around the souslik burrow, COLONY—plots in the area of the souslik colony (determined by KDE95%) without taking into account areas near the burrows, CONTROL—plots located outside the souslik colony within the remaining area of the studied grassland (for details see the methods). In a pairwise comparison of the frequency of mole mounds, all differences were statistically significant (p < 0.05) in each year of the study, except from BURROW-COLONY in 2020. The figure was generated in SPSS v24.0 (https://www.ibm.com/products/spss-statistics) and CorelDRAW Standard 2020 (https://www.corel.com).

The 3-year field monitoring showed that the coexistence of the souslik and the mole in a relatively homogeneous area of the airfield’s grassland is not random and there is avoidance in the space between these species. In the first zone (BURROW), signs of the presence of moles were found rarely, on average in 6% of the study plots. These values were low (range: 4–11%) in all years of the study. In the second zone (COLONY), the mole mounds were found on average in 18% of the study plots. These values were relatively constant over the 3-year study and ranged between 15 and 20%. The statistical analysis showed that the frequency of the presence of the mole mounds in the zones BURROW and COLONY differed significantly in two of three study years (p = 0.002 in 2018 and 2019, p = 0.154 in 2020) (Fig. 6). The spatial avoidance of the mole and the souslik is most clearly shown by the results obtained for the CONTROL zone. In this zone, signs of the presence of moles were found in 44% (range: 36–56%) of the study plots, which indicated that the mole is a common species in the airfield. The values obtained for the CONTROL were significantly different from the results in the BURROW (p < 0.0001 in all years) and COLONY (p = 0.004 in 2018, p < 0.0001 in 2019 and 2020) zones (Fig. 6).

### Analysis of aerial imagery

**Figure 7:**
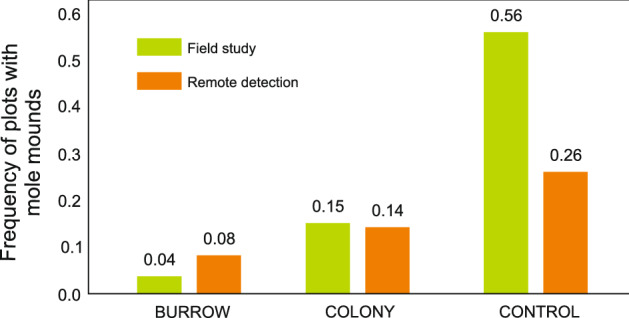
Frequency of plots with mole mounds assessed in the field study and by remote sensing. The clear avoidance of sousliks and moles near the souslik burrows and home range of the souslik colony was shown by both methods, but the remote methods indicated a lower level of occurrence of the mole in the control plots. All differences between the zones were statistically significant (p < 0.05) in the pairwise comparison of the frequency of the mole mounds in the field study. In the case of the remote detection, there were statistically significant differences in the BURROW-CONTROL and COLONY-CONTROL pairs (p < 0.05). The figure was generated in SPSS v24.0 (https://www.ibm.com/products/spss-statistics) and CorelDRAW Standard 2020 (https://www.corel.com).

Data on the presence of mole mounds obtained from the aerial imagery showed a similar relationship between the mole and souslik occurrence to that shown by the field data described above. Mole mounds were identified in 8% of the study plots in the BURROW zone and in 14% of the plots in the COLONY zone. These values were within the range noted in the field studies (Fig. 6). However, a substantially lower value was obtained for the CONTROL zone, where the mole mounds were identified only on 26% of the study plots (Fig. 7), while the field data indicated a range of 36–56% (Fig. 6). Despite this lower value for the third zone, the statistical analysis showed significant differences in the BURROW-CONTROL (p < 0.0001) and COLONY-CONTROL pairs (p = 0.025) (Fig. 7). Self-trained Faster-RCNN model for IoU ≥ 0.5 achieved average precision (AP) of 0.6845 and an F1-Score of 0.7993 with Recall of 0.8236.

### Consequences for souslik monitoring

**Figure 8:**
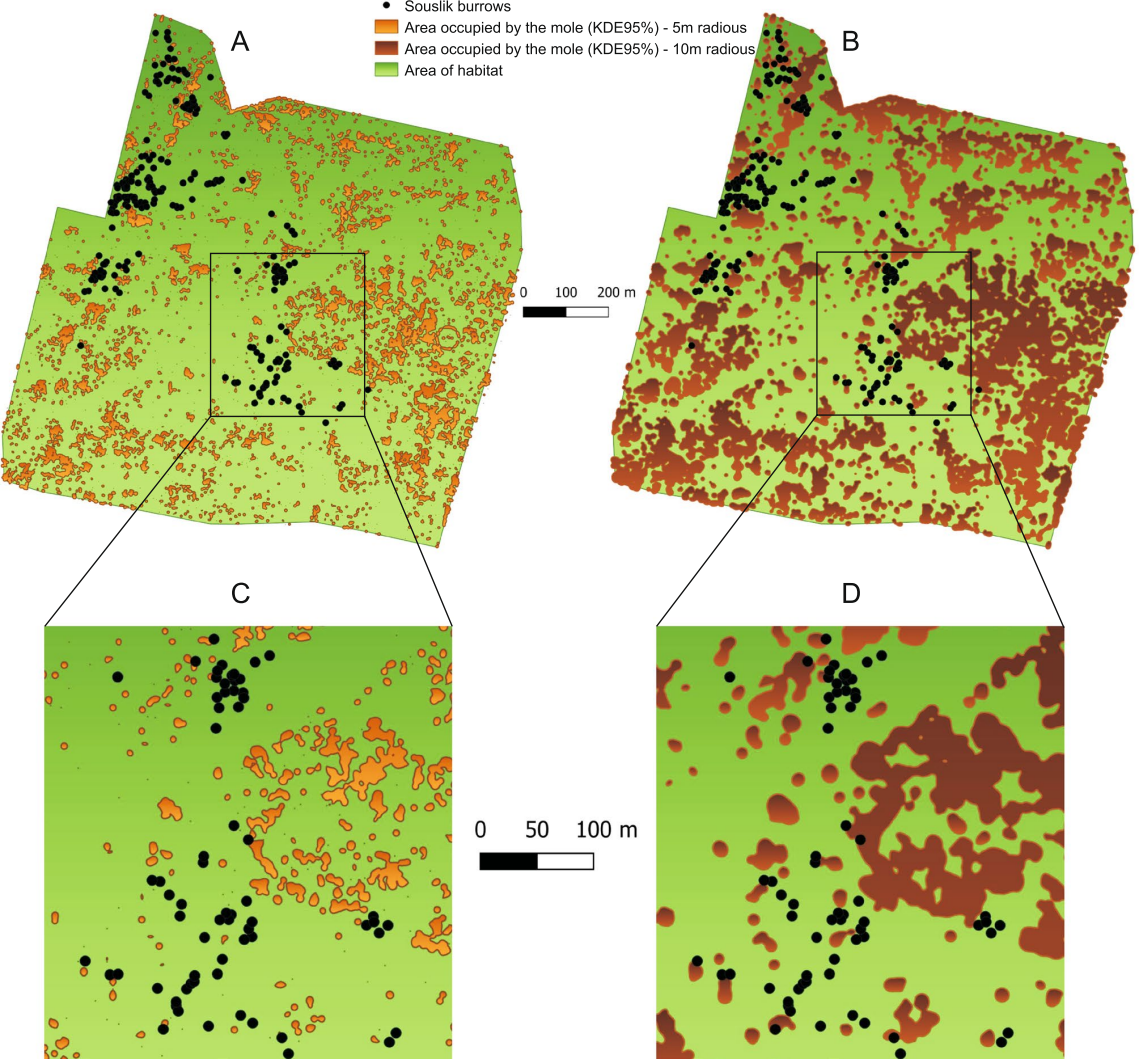
Location of 228 souslik burrows in 2019 and areas occupied by the mole designated on the basis of a 5 m radius for the 95% kernel (**A**) and a 10 m radius for the 95% kernel (**B**). For a more precise visualization, the enlarged central parts of the area are shown (**C**,**D**). The locations of sousliks’ burrows were determined in the field studies, and the area occupied by the mole was determined by the analysis of aerial images. There is a clear spatial separation of these two species: the souslik burrows overlap with the area occupied by moles only in 0.9% of the cases in figure (**A**) and in 8.7% of the cases in figure (**B**). The figure was generated in Quantum GIS v.3.4.5 (https://qgis.org) and CorelDRAW Standard 2020 (https://www.corel.com).

The area occupied by the mole determined by the analysis of aerial imagery was approximately 15 ha for a radius of 5 m and approximately 40 ha for a radius of 10 m (14% and 38% of the study area, respectively). This potentially limits the habitat suitable for the souslik from 105 to 89.6 ha or 64.8 ha, depending on the applied radius of 5 or 10 m (Fig. 8). In fact, the souslik burrows were located usually outside the mole-occupied area: only 2 out of 228 (0.9%) sousliks had their burrows in the area occupied by the mole for parameters presented in Fig. 8A. When wider parameters of the mole area boundaries (10 m) were established, the percentage of overlapping burrows increased to the value of 8.7% (Fig. 8B). This value is similar to the results of the field studies presented above, where mole mounds in the vicinity of souslik burrows were found in 4–11% of cases (Fig. 6).

The knowledge that the area of the optimal habitat for sousliks is dependent on the distribution of moles can be used in the designs of souslik monitoring and limits labour consumption in such fieldwork. Traditionally, monitoring is performed by observers walking on transects. Assuming that the observer moves at an average speed of 3 km h^−1^ and is able to inspect visually a 5 m wide strip (2.5 m to the right and the left of marching line), it is possible to calculate that inspection of 1 ha takes 40 min, and the route that the observer has to walk is 2 km. This means that reducing the monitoring area by 40 ha saves about 27 h of fieldwork and excludes the need to walk the 80 km route, i.e. at least 4 days of fieldwork for one person.

## Discussion

Our study combining field data and aerial imagery analysis clearly showed that the spotted souslik avoids close coexistence with another burrowing species, i.e. the European mole, in the period of low population abundance. This is the first study on this subject described in the available literature, as attention has been paid mainly to other parameters of the habitat so far^14,18,20^. The present results can (1) make a new contribution to the knowledge of the ecology of burrowing mammals and their interspecies relationships, (2) contribute to better designs of conservation and assessment of the quality of habitats of endangered burrowing mammals, and (3) indicate new possibilities of using remote sensing and deep learning methods in ecology and conservation. Below we will try to address each of these issues.

The interaction between underground animals is not a new idea in ecology (e.g.^22^); however, this issue has not been analyzed for the mole and the souslik so far. This was probably related to the fact that the potential negative or positive relationships between these species are not intuitively obvious. The spatial distribution of underground tunnels of these animals is completely different: the mole builds an extensive network of horizontal tunnels close to the ground surface, while the souslik usually builds one deep nest burrow with a vertical entrance and possibly a small number of shallow safety burrows near the nest burrow. Moreover, the food preferences of the souslik and the mole differ, i.e. the former is mainly a herbivore, while the latter is an obligatory predator. There are also clear differences in the annual cycle: the mole is active all year round, and the souslik hibernates in an underground nest for about half a year from October to March. Thus, it seems that the emergence of competitive relationships between these two species is unlikely. Our study shows, however, that these species avoid each other in space, which raises the question of the mechanism of this relationship. Based on the knowledge of the biology of both species, some hypothetical mechanisms can be proposed.

Although they are colonial animals, sousliks inhabit burrows alone (except for mother and offspring) and they have a strong behavioural trait of a negative reaction to the presence of other animals in their burrows and their close vicinity^14,23^. The negative reaction to other sousliks is a reflection of the intraspecific competition in the population and the territoriality of individuals. It is regulated by odour signals and the social structure of the population^30,31^. Koshev^32^ described aggressive reactions of free-ranging European sousliks to other vertebrate species that appeared near burrows: towards the reptile *Lacerta trilineata*, the bird *Corvus frugilegus*, and the mammal *Mustela nivalis*. Theoretically, the mole can get into the souslik’s burrow unintentionally when digging new tunnels. For souslik, the presence of moles in their nest burrow means a violation of its strictly defended territory and is probably a highly stressful episode. It can therefore be assumed that sousliks should choose places outside areas of frequent occurrence of other burrowing mammals to set up a nest burrow.

It remains an open question whether avoidance of areas where the mole is often present may be important for the souslik during winter hibernation. Theoretically, the presence of moles in souslik burrows during hibernation may disturb this process and cause waking up and energy-consuming increases in metabolism, which may reduce winter survival. It is also unknown whether the mole can be a predator for the souslik during winter hibernation. Remains of rodent species were found in the digestive tracts of moles^33^; therefore, at least theoretically, the mole may use such a food source. On the other hand, remains of vertebrates, including the remains of moles, were sometimes found in the stomachs of sousliks^18^. The relationship between the souslik and the mole may therefore be more complex and require further research focused on this issue. It is possible that the moles can avoid the souslik colonies as well. This scenario seems also realistic, since the moles home ranges are likely much more dynamic than that of sousliks, that likely benefit from dwelling within an existing colony of the conspecifics.

The spotted souslik protection requires the designation of special areas of conservation^16^. A number of various conservation activities are also routinely undertaken for this species, including regular monitoring of the population size, habitat monitoring, mowing, reduction of predation risk, and application of more invasive methods such as reintroduction. Similar activities are also performed for a closely related species, i.e. the European souslik *Spermophilus citellus*, in Europe. Importantly, in the current guidelines of souslik conservation, the issue of the competition with other species and its impact on spatial distribution is not considered. In turn, there is evidence in the literature that interspecies interactions may be important for the souslik population^21^. In periods of low abundance, when the survival of the population is at risk, the sousliks may have different habitat preferences than in periods of the abundant population^20^. It seems, therefore, that nowadays, when the souslik most often forms small populations, more attention should be paid to a wider range of factors and threats that may determine longer term population trends or the health condition, survival, and abundance of their colonies.

Our study indicates that, in the period of low population abundance, the presence of other burrowing species may be an important factor determining the distribution of sousliks. This observation shows that in addition to the assessment of the area and condition of the habitat the presence of other potentially competitive species should also be taken into account in the analysis of population survival. In such a case, the actual area of habitats suitable for sousliks in a given location may turn out to be much lower than assumed. In our study area, the habitat suitable for the souslik was reduced from 105 ha to approx. 65 ha, i.e. by nearly 38%, but it probably is even smaller (compare Fig. 8). This observation has consequences for improvement of the reintroduction methods of sousliks (or other burrowing mammals), which are constantly of scientific interest^20,34,35^. Our results indicate that the reintroduction of sousliks should be carried out in places where there is the lowest probability of competition for resources including even shelter or space with other burrowing species and where adequate space for the settlement of the population is ensured.

So far, investigations of the distribution of small burrowing mammals have been based on laborious field studies involving site inspections by trained observers (e.g.^36–38^). Our results show that, in certain conditions, high-resolution imagery can be successfully used to support studies of the distribution of such animals. As reported by other authors (e.g.^7,10,12^), however, such animals must produce clear signs of their presence in the environment. Evidence of the presence of the European mole, i.e. mounds of soil, in short vegetation habitats has shown that remote sensing can detect moles and their area of occupancy successfully. The advantage of these markers of the presence of moles is that the mounds are redundant and quite durable and can be visible in the environment for up to several months.

By combining field research and remote sensing, it is also possible to study more sophisticated ecological issues, e.g. interspecies interactions. In this work, the remote estimation of the distribution of moles facilitated estimation of the actual habitat available to the souslik and excluded areas with the lowest probability of its occurrence. As a result, the population may be monitored more economically. Since the conservation guidelines recommend monitoring souslik populations by means of laborious inspections of transects, the indication of areas with no burrows may significantly reduce the amount of fieldwork without negative consequences for the accuracy of results. Some areas of the souslik occurrence are large, e.g. Świdnik (105 ha) or Pastwiska nad Huczwą (150 ha), and every 10 ha to be monitored means one day's work for one observer (according to the calculations presented in the results). Our study showed that when the area of the occurrence of moles is excluded from the monitoring (Fig. 8), the error in estimating the size of the souslik population will be relatively small (0.9–8.7%). At the same time, the time devoted to the research can be limited by 14% or 38%, respectively. This suggests that our method can contribute to improved monitoring and management of these protected species, especially that souslik monitoring requires considerable research effort and has to be carried out twice a year.

However, mole mounds may be underestimated by remote sensing, which can be seen in Fig. 7. Small mole mounds that are easily identified during field research may not be noticed by remote sensing. Such underestimation does not constitute a critical threat to the determination of the mole area according to the scheme shown in Fig. 8, since its marks are highly redundant. However, since there is currently little research on this subject, we recommend combining field research and remote sensing in assessments similar to ours. Finally, it is worth noting that, for a better understanding of the issue of the interactions between souslik and other burrowing species, it is advisable to use another remote sensing technique—telemetry. Telemetry studies are successfully conducted in Bulgarian souslik populations^34^ and their combination with studies of habitat selectivity dependent on other burrowing species may provide new and valuable insight into this issue.

## Data Availability

The datasets generated during the current study are available from the corresponding author on reasonable request.
